# A New Method for Assessing Content Validity in Model-Based Creation and Iteration of eHealth Interventions

**DOI:** 10.2196/jmir.3811

**Published:** 2015-04-15

**Authors:** Nancy Kassam-Adams, Meghan L Marsac, Kristen L Kohser, Justin A Kenardy, Sonja March, Flaura K Winston

**Affiliations:** ^1^Center for Injury Research and PreventionChildren's Hospital of PhiladelphiaPhiladelphia, PAUnited States; ^2^Perelman School of MedicineUniversity of PennsylvaniaPhiladelphia, PAUnited States; ^3^Centre of National Research on Disability and Rehabilitation Medicine and School of PsychologyUniversity of QueenslandBrisbaneAustralia; ^4^University of Southern QueenslandSpringfieldAustralia

**Keywords:** telemedicine, methods, stress disorders, post-traumatic, child, secondary prevention

## Abstract

**Background:**

The advent of eHealth interventions to address psychological concerns and health behaviors has created new opportunities, including the ability to optimize the effectiveness of intervention activities and then deliver these activities consistently to a large number of individuals in need. Given that eHealth interventions grounded in a well-delineated theoretical model for change are more likely to be effective and that eHealth interventions can be costly to develop, assuring the match of final intervention content and activities to the underlying model is a key step. We propose to apply the concept of “content validity” as a crucial checkpoint to evaluate the extent to which proposed intervention activities in an eHealth intervention program are valid (eg, relevant and likely to be effective) for the specific mechanism of change that each is intended to target and the intended target population for the intervention.

**Objective:**

The aims of this paper are to define content validity as it applies to model-based eHealth intervention development, to present a feasible method for assessing content validity in this context, and to describe the implementation of this new method during the development of a Web-based intervention for children.

**Methods:**

We designed a practical 5-step method for assessing content validity in eHealth interventions that includes defining key intervention targets, delineating intervention activity-target pairings, identifying experts and using a survey tool to gather expert ratings of the relevance of each activity to its intended target, its likely effectiveness in achieving the intended target, and its appropriateness with a specific intended audience, and then using quantitative and qualitative results to identify intervention activities that may need modification. We applied this method during our development of the Coping Coach Web-based intervention for school-age children.

**Results:**

In the evaluation of Coping Coach content validity, 15 experts from five countries rated each of 15 intervention activity-target pairings. Based on quantitative indices, content validity was excellent for relevance and good for likely effectiveness and age-appropriateness. Two intervention activities had item-level indicators that suggested the need for further review and potential revision by the development team.

**Conclusions:**

This project demonstrated that assessment of content validity can be straightforward and feasible to implement and that results of this assessment provide useful information for ongoing development and iterations of new eHealth interventions, complementing other sources of information (eg, user feedback, effectiveness evaluations). This approach can be utilized at one or more points during the development process to guide ongoing optimization of eHealth interventions.

## Introduction

### Overview

The advent of eHealth interventions to address psychological concerns and health behaviors has created new opportunities and new challenges. Some eHealth interventions are adaptations of established face-to-face interventions; many are created from scratch as electronically delivered interventions. In either case, they provide the ability to optimize the effectiveness of intervention activities and then deliver these activities consistently to a large number of individuals in need.

Ideally, development of any intervention (whether the intervention is delivered electronically or in-person) begins with a clearly delineated program theory or model of change that is grounded in empirical evidence and clinical experience [1,2]. In such a model, intervention activities target specific mechanisms (psychological or behavioral processes) in order to produce desired modifications in health or behavioral outcomes. The use of a theoretical model to guide development of an eHealth intervention appears to be associated with effectiveness. A recent meta-analysis of 85 studies of eHealth interventions for health behavior change found that interventions that made greater use of theory (ie, linking theoretical constructs to intervention techniques) had larger effect sizes [3].

### Definition of Content Validity and Adaptation for eHealth Interventions

The concept of content validity originates in the arena of psychological and educational instrument development. Content validity of an assessment instrument is one aspect of construct validity [4,5] and has been defined as “the degree to which elements of an assessment instrument are relevant to and representative of the targeted construct for a particular assessment purpose”, page 238 [5]. In this definition, the relevant elements of an instrument may vary based on the method and purpose of the assessment and include item wording as well as the way in which stimuli are presented, how instructions are given, and which situations are sampled [5]. Content validity is conditional rather than an inherent trait of an assessment instrument; it is assessed with regard to a particular purpose or aim of assessment, and a particular targeted population [5]. Quantitative and qualitative indicators derived from expert review of an instrument’s content validity can be useful in identifying missteps and honing content during the development phase of an assessment instrument [5,6].

Our definition of content validity for eHealth builds upon these well-established attributes of content validity in instrument development. We define the content validity of an eHealth intervention as the extent to which its component intervention activities are relevant to the underlying construct (ie, program theory) and likely to be effective in achieving a particular intervention purpose in a specific intended population. We therefore suggest three core dimensions for expert review: relevance, likely effectiveness, and appropriateness for a specific audience. The first dimension, *relevance*, is the extent to which an intervention activity is pertinent to its intended intervention target as defined in the program theory or model of change, that is, “Is this arrow aimed in the right direction?” The second dimension, *likely effectiveness*, is the extent to which evidence, theory, and expert judgment would suggest that this specific activity would successfully modify the intended intervention target, that is, “Is this arrow likely to hit the target?” The third dimension is the extent to which the activity is *appropriate for a specific intended audience*, which may be defined by age, culture, or other factors.

### Rationale for Considering Content Validity of eHealth Interventions

There are several compelling reasons to attend carefully to content validity in the development of eHealth interventions. When intervention content is developed based on a clearly delineated program theory and model of change, not only is the eHealth intervention more likely to be effective, its use and evaluation also advances understanding of the psychological or behavioral processes in which one is trying to intervene [7,8]. However, after the developer of an eHealth intervention has articulated a model of change/program theory, the next steps are fraught with challenges, including a multitude of choices in the design and delivery of intervention activities. A formal assessment of content validity can be a key checkpoint in the design of actual intervention activities to ensure that these activities and processes match the underlying program theory and change model that they are intended to operationalize. Electronically delivered intervention programs (to an even greater extent than manualized in-person interventions) deliver a set of pre-determined and highly observable activities, thereby facilitating review of specific activities during the development process. Results of content validity assessment can be used to hone an eHealth intervention during its development or to better understand unexpected variations in the performance of an existing intervention. Given the expense of developing eHealth interventions, assessing content validity early in the development process (eg, at the storyboard stage) could be cost-effective by increasing the likelihood that costly further development will lead to an effective intervention.

Undoubtedly, most intervention developers strive to achieve this sort of validity and informally assess the extent to which they have succeeded. However, to our knowledge no systematic process for assessing content validity of eHealth interventions has been proposed. Thus, the aims of this paper are to define content validity as it applies to model-based eHealth intervention development, present a feasible method for assessing content validity in this context, and describe the implementation of this method during the development of an intervention. Based on the definition of content validity for eHealth proposed above, we designed a practical method for assessing the content validity of an eHealth intervention, and applied this methodology during our development of the Coping Coach Web-based intervention for school-age children. Formal review by external experts allowed us to evaluate the extent to which intervention activities matched the model of change and program theory upon which we based our intervention development.

## Methods

### Procedures for Expert Review of Content Validity

We propose a straightforward, systematic approach to obtaining expert review of the content validity of an eHealth intervention. This approach assumes that the eHealth intervention in question has been created based on an explicit program theory or model of change or that an appropriate theory/model can be applied (even retrospectively) to the existing intervention content. Our approach consists of the following steps:

- Step 1 is to specify key intervention targets that this e-Health intervention is intended to address.
- Step 2 is to delineate specific activity-target pairings, by defining discrete intervention activities that address each target. These may not be one-to-one relationships; a target may be addressed by more than one activity or vice versa. In this context, an intervention activity is defined as a meaningful set of user actions or experiences that can be clearly linked to one or more targets. It is important to be able to describe each activity so that expert reviewers understand exactly which intervention content is included.
- Step 3 is to populate the Content Validity Survey Tool with each intervention activity-target pairing. The Survey Tool includes scales for relevance, likely effectiveness, and appropriateness for a specific intended audience (see Multimedia Appendix 1).
- Step 4 is to recruit experts who were not involved in the development of the eHealth intervention and gather survey data using the Content Validity Survey Tool. Expert reviewers should each have relevant content knowledge; disciplinary and geographic diversity across the set of reviewers can provide useful balance [5,9]. The ideal number of reviewers has been suggested as 8-12 for an initial stage review of content validity, and 3-5 (who may be a subset of the original group) for a secondary or follow-up review [6]. Expert reviewers must be provided with access to the best current version of the eHealth intervention (eg, storyboard, text script, online access to the intervention) and asked to complete an online or emailed copy of the Content Validity Survey Tool created in Step 3.
- Step 5 is to analyze results and refine the intervention as needed. Results should be analyzed quantitatively (eg, via calculation of content validity indices) and qualitatively (eg, via examination of narrative comments from expert raters). Use these findings to identify potentially problematic activities and to hone the intervention as needed. Depending on the stage of development of the intervention, the development team may elect to remove or revise potentially suboptimal intervention activities immediately or in a future iteration of the intervention.

Based on prior literature regarding content validity in instrument development, we propose both item-level and scale-level content validity indices (I-CVI and S-CVI/AV) as quantitative indicators of acceptable content validity [6]. For eHealth interventions, an “item” is an intervention activity-target pairing, and the “scale” is a set of activity-target pairs. The I-CVI is the proportion (0.0 to 1.0) of expert reviewers who rate an item as 3 or 4 on a 4-point scale. The S-CVI/AV is the average of all I-CVIs for a set of items [6]. Polit et al [6] proposed standards for content validity based on a review of the literature and examination of the quantitative properties of alternate content validity indices. We propose to adopt these standards for eHealth, such that a set of eHealth intervention activities can be said to have excellent content validity if all I-CVIs are at least .78 and the S-CVI/AV is at least .90.

Utilizing the results of content validity assessment to hone and improve an eHealth intervention will always involve both quantitative indicators and the considered judgment of the development team. For example, if a quantitative indicator such as the I-CVI indicates problems with a specific activity-target pair, the next steps for the development team depend on the nature of the problem identified. If an activity is rated as not relevant to its intended target, the development team may consider removing it or undertaking a major revision. On the other hand, if an activity is rated as relevant but not likely to be effective, the development team should consider whether there is a way to alter or enhance the activity to increase its likely effectiveness. Narrative comments provided by expert reviewers as part of the Content Validity Survey can be helpful, and follow-up interviews to elicit additional details about specific concerns may be useful. Developers may also need to take into account whether an activity is rated as relevant/likely effective for some, but not all, of its intended targets.

### Application of this Method to the Coping Coach Intervention

#### Description of Coping Coach

Coping Coach is an eHealth intervention designed to prevent or reduce posttraumatic stress and associated negative impacts on health-related quality of life in young people aged 8-12 years old who have experienced different types of acute, single-incident traumatic events [10]. The Coping Coach intervention is structured as an interactive game with a storyline. Intervention activities include skills practice and interactions with game characters as the child user progresses through three levels of the game. The program theory that underlies our development of the Coping Coach intervention is presented in detail elsewhere [10,11] and described briefly here. Grounded in the empirical literature on posttraumatic stress etiology [12-14], we first identified four proximal goals for users of the Coping Coach intervention: (1) identify emotional reactions after trauma, (2) build cognitive re-appraisal skills, (3) reduce use of avoidance coping strategies, and (4) increase social support seeking. For each of these goals, we drew from the empirical literature on intervention for posttraumatic stress and anxiety in children [15-17] to delineate more specific actionable intervention targets and then worked closely with a Web/game developer team to craft intervention activities to address these targets.

#### Application of 5-Step Content Validity Process to Coping Coach

Step 1 (specify key intervention targets) was integrated throughout the development process. As described above, we developed the intervention based on a program theory in which we identified 13 specific intervention targets (2-5 targets for each of the four proximal goals) to address key mechanisms for prevention of posttraumatic stress.

In Step 2 (delineate activity-target pairings), we delineated 11 discrete intervention activities; each activity addressed one or more of the 13 intervention targets, resulting in a total of 15 activity-target pairs. Table 1 presents each intervention activity with the target(s) it was intended to address. For the Coping Coach intervention, the process of delineating activity-target pairings was straightforward, as our intervention development process began with a careful definition of intervention targets, and each activity was designed to address one or more of these targets. When an intervention has not been developed explicitly in this manner, Step 2 may require additional effort such as mapping activity-target pairings via a consensus process among key members of the development team and/or evaluating interrater reliability in matching activities to targets.

For Step 3 (populate survey tool), we created a Content Validity Survey Tool with three ratings for each of the 15 activity-target pairs. For each activity-target pairing, experts rated the intervention activity’s relevance, likely effectiveness, and age-appropriateness, using a 5-point Likert-type scale (0-4), as follows: (1) *Relevance* (the extent to which this specific intervention activity is pertinent to the intended intervention target) with 0 defined as “Irrelevant/Extraneous to this target” and 4 defined as “Central/Key/Essential to this target”; (2) *Effectiveness* (likelihood that this specific activity will successfully modify the intended intervention target), with 0 defined as “Not likely to be effective” and 4 defined as “Very likely to be effective”; and (3) *Appropriateness for intended audience*, which, in the case of Coping Coach, was defined as age-appropriateness (extent to which the language, content, and nature of activities was clear, easy to understand, and developmentally appropriate for children age 8-12 years), with 0 defined as “Inappropriate/Unsuitable for 8-12” and 4 defined as “Language/nature of activities appropriate for 8-12”. The survey form included screen shots from the intervention to help orient expert reviewers to the activity they were rating. A copy of the Content Validity Survey Tool template is available in Multimedia Appendix 1.

For Step 4 (recruit experts and gather survey data), an international set of experts was invited to participate in rating the Coping Coach intervention. Experts were selected based on their knowledge and expertise regarding children’s coping and adjustment after potentially traumatic events, traumatic stress prevention, culturally sensitive child interventions, or development of Web-based interventions. We provided each expert with a username and password to access the Coping Coach intervention online and encouraged them to play through the entire game at least once as a child user would. We also provided a full text transcript of all intervention elements and activities. The Content Validity Survey Tool was provided as a Word document and sent to experts via email; experts completed their ratings within this document and returned the document via email. Expert reviewers were asked to complete the Content Validity Survey Tool and to provide additional comments on any specific activity or on the intervention as a whole. Expert review of an intervention does not constitute human subjects research, and thus no Institutional Review Board or ethics board approval process was relevant or required.

For Step 5 (analyze results and hone intervention), we first calculated the I-CVI for each activity-target pair on each dimension; the I-CVI is the proportion of reviewers who gave a rating of 3 or 4 on the 5-point scale (0-1-2-3-4) used in this version of the Survey Tool. We then calculated the S-CVI/AV for each dimension (relevance, likely effectiveness, and age-appropriateness) as the average of all I-CVIs for that dimension. In this case the I-CVI is a slightly more conservative indicator of expert consensus than described by Polit et al, because the survey tool utilized for ratings of Coping Coach used a 5-point scale rather than a 4-point scale. We also examined additional narrative comments from the expert reviewers. These data, in conjunction with feedback from child users and their parents [10] and the results of a pilot randomized trial [11], are now being used to hone and improve the next iteration of the Coping Coach intervention.

## Results

### Overview

A total of 15 experts (from the United States, Australia, United Kingdom, Netherlands, and Switzerland) were invited to participate via email; all 15 agreed to participate and provided ratings. All experts were independent, that is, not involved in the development of the intervention. Each expert was an active clinical researcher (12 psychologists and 3 psychiatrists) with at least 5 years of experience in this field and relevant content expertise. Thirteen experts provided ratings within approximately 2 months as requested; 2 experts required additional time due to other commitments but eventually provided ratings. There were very few incomplete ratings (only 2 of 225 ratings for relevance, 3 of 225 ratings for effectiveness, and 5 of 225 ratings for age-appropriateness were missing).

### Quantitative Indicators of Content Validity

Table 1 shows the I-CVI for each intervention activity/target pairing, based on expert ratings from the Content Validity Survey Tool.

**Table 1:** Intervention activities and intervention targets for each of four proximal goals of the intervention, with item-level Content Validity Index for each activity-target pairing.

Intervention activity		Intended intervention target(s) for this activity	Item-level Content Validity Index		
			Relevance of activity to target	Likely effectiveness of activity	Age appropriateness of activity
**A. Proximal goal: Identify emotional reactions (EM)**					
	1. Player creates faces by manipulating the eyebrows and mouth to match specified feelings in Face-O-Matic Machine.	EM1: Child will identify and name basic feelings/ emotions.	.80	.60	.73
	2. Player helps the townspeople identify how they were feeling at the time of a potentially traumatic event, and how they are feeling now.	EM2: Child will identify feelings/ emotions associated with a potentially traumatic experience, and how these feelings may change over time.	.93	.93	.93
	3. Player identifies own feelings with the help of the townspeople.	EM3: Child will identify their own feelings associated with a potentially traumatic experience, and any changes in these feelings over time.	.93	.86	1.0
**B. Proximal goal: Build cognitive re-appraisal skills (CR)**					
	4. Player watches/ listens to conversation between General Malaise and the Coping Coach about Think→ Feel→ Act.	CR1: Child will recognize connections between thoughts (appraisals), feelings, and behavior.	1.0	.73	.73
		CR2: Child will recognize helpful/ unhelpful trauma-related thoughts/ appraisals and see appraisals as something that can be modified.	.93	.73	.67
	5. Player helps Jack and Jayla understand their thoughts and feelings and then helps Jack/Jayla change unhelpful thoughts in order help them to feel better.	CR3: Child will identify helpful/ unhelpful trauma-related appraisals.	1.0	.87	.87
		CR4: Child will use cognitive restructuring to modify unhelpful appraisals.	1.0	.80	.79
	6. Player identifies own helpful/unhelpful thoughts by selecting whether statements are “like me” or “not like me”, and player’s helpful thoughts guide the airship upwards.	CR5: Child will identify their own helpful and unhelpful thoughts/ appraisals and apply cognitive restructuring to modify own unhelpful appraisals.	.93	.80	.86
**C. Proximal goal: Reduce use of avoidance coping strategies (AV)**					
	7. Coping Coach describes Avoidance and Approach strategies. Player helps townspeople identify pros/cons of avoidance, sees 2 people modeling approach strategies, and helps 2 people replace avoidance with approach strategies.	AV1: Child will identify pros/ cons of avoidance and approach strategies for trauma-related fears/situations.	1.0	.93	.87
		AV2: Child will approach trauma-related fears situations safely and minimize reliance on avoidant coping strategies.	1.0	.87	.87
	8. Sorting activity – player fixes weather machine by correctly identifying pros/cons and impact of using avoidance or approach strategies for trauma-related fears/ situations.	AV1: Child will identify pros/ cons of avoidance and approach strategies for trauma-related fears/ situations.	.87	.93	.80
		AV2: Child will approach trauma-related fears situations safely and minimize reliance on avoidant coping strategies.	.80	.80	.79
**D. Proximal goal: Increase social support seeking (SS)**					
	9. Player gives and receives help to/ from the townspeople and General Malaise.	SS1: Child will ask for help and build support network by providing help to others.	1.0	.80	1.0
	10. Player completes logbook pages to identify “People Who Can Help Me” and “Ways That People Can Help Me”.	SS2: Child will identify members of their support network and what type of support network can offer.	1.0	.93	.93
	11. Player collects coins scattered throughout the worlds – six of these coins have tips for social support seeking.	SS3: Child will increase strategies for asking for help/ social support.	.86	.79	.86

The S-CVI/AV for ratings across all activities was excellent for relevance (.94), although not quite at this standard for likely effectiveness (.82) or age-appropriateness (.85). Examining the quantitative indicators at a more granular level, the I-CVIs for likely effectiveness and age-appropriateness were excellent (≥.78) for nearly all activity-target pairings. However, we identified two activities (encompassing three activity-target pairs) with I-CVIs below .78. Intervention activity 1 (Face-O-Matic Machine activity) and intervention activity 4 (conversation between Coping Coach and General Malaise character), had excellent ratings for relevance but had I-CVIs of .60 to .73 for likely effectiveness or age-appropriateness. Reviewers also provided narrative comments to explain their concerns and/or suggest alternate approaches. Based on these I-CVIs and review comments, these two activities are under review to determine whether they should be retained, removed, or modified in the next iteration of the intervention.

### Narrative Comments From Expert Reviewers

Beyond quantitative ratings, for many intervention activities the reviewers’ narrative comments were helpful in understanding both strengths and potential gaps in this iteration of the Coping Coach intervention. Reviewers commented on likely mechanisms of action, for example, “One of strongest sections, teaches link between thoughts, feelings, and actions well, and good in identifying concrete thoughts” and “Interactive nature of the exercise and the fact that it doesn’t ‘sugar coat’ that there are some positives to avoidance is useful as it makes it realistic for kids”. Reviewers also highlighted ways to extend or improve current intervention activities to better achieve key targets, for example, “Perhaps also discussing what a child’s behavioral reactions may be when sad, angry, worried, etc (for example, crying, stamping feet, churning stomach) may offer them more of a chance to identify their feelings”.

## Discussion

### Principal Findings

This project demonstrated that assessment of content validity was straightforward and feasible to implement and that results of this assessment can provide useful information for ongoing development and iterations of new eHealth interventions. Expert ratings on the Content Validity Survey Tool demonstrated variability, suggesting that response options were appropriately scaled and anchored to capture useful gradations in expert judgment about the content validity of specific intervention activities.

Especially for components believed to be key to intervention outcomes, assessment of content validity could reduce the number of iterations needed to produce an effective eHealth intervention. The clear articulation of a model of change and program theory, and content validity assessment to ensure that intervention activities match their intended targets, may be especially important in the development of eHealth interventions that are created from scratch, that is, that are not Web-based adaptations of an existing well-established face-to-face intervention [10,18]. However, content validity assessment may also be beneficial to ensure that well-established face-to-face interventions are successfully translated for Web-based/digital delivery. The translation of in-person treatment components (eg, exposure to address anxiety symptoms) is not always straightforward.

Expert ratings of content validity can be an important complement to other sources of information. Depending on the point in the development cycle when content validity is assessed, an intervention development team may need to weigh information regarding suboptimal content validity of specific activities in the context of user feedback, effectiveness evaluations, and other contextual considerations to determine appropriate action. One possibility is immediate refinement or removal of potentially problematic activities; another option is ongoing monitoring of these activities in terms of user engagement or effectiveness. In the case of Coping Coach, expert review affirmed the relevance of all rated activities but identified potential gaps in the likely effectiveness and age-appropriateness of two intervention activities. This information will be utilized in conjunction with user feedback and results of a pilot randomized trial to make decisions about optimizing Coping Coach intervention activities.

Implementation of this method revealed a number of lessons regarding the process and timing of assessing content validity. Regarding process, we learned that thoughtful judgment by the development team is required to define intervention activities at an appropriately granular level, that is, with just enough specificity for meaningful evaluation by expert reviewers. Thoughtful judgment is also required to identify which activity-target pairs merit assessment of content validity. In the case of Coping Coach, we chose to structure the survey with each intervention target paired with the one or two activities that addressed that target most directly. However, some intervention targets are addressed at least indirectly by additional activities and a longer survey form could have asked expert raters to assess all such activity-target pairings. One potential outcome of Steps 1 and 2, or of feedback received during expert review (Step 4), is that intervention developers may realize that they have not adequately specified intervention targets or the intended match between intervention activities and intended targets. If this occurs, it can be seen as an important reminder for the development team, aided by expert consultation if necessary, to revisit and clarify the program theory and model of change that underlie the eHealth intervention. Clarity in this regard is likely to be helpful not only in intervention design, but also in promoting effectiveness of the intervention.

Regarding timing, we sought expert review of content validity at a point in the development process when we had already created a functional online intervention, piloted this intervention with child users, and initiated a pilot randomized trial. The advantage of this timing is that experts saw a fully developed version of the intervention activities and could fully grasp our intended design. There would be different advantages to seeking expert review earlier (with storyboards or functional prototypes) or at multiple points in the development process, namely the ability to iteratively revise an earlier draft of the intervention based on content validity assessment.

### Limitations

There are several limitations of this project that suggest future research directions. First, we suggest the application of quantitative indicators (the I-CVI and S-CVI/AV), and threshold levels for those indicators, which are based in content validity research for the development of psychological measures. While we believe this is a reasonable place to start, additional research is needed to document the range of I-CVI and S-CI/AV results in the development of a variety of types of eHealth intervention activities, and the relationship of these ratings to improved performance of eHealth interventions. Such research would also help to assess the reliability and validity of the proposed Content Validity Survey Tool itself, as this was beyond the scope of the current study. It is important to note that we implemented this content validity approach for an eHealth program with a “tunnel” design, in which all users are required to participate in all activities. Because eHealth interventions vary in the extent to which every user is directed to participate in the same set of activities, assessment of content validity for more complex branching structures, or for interventions that allow free exploration of a set of activities, may require some adaptation of our method. The proposed method for content validity targets three domains: relevance, likely effectiveness, and appropriateness for a specific audience. As the tool becomes more widely used, a need might be recognized for additional domains. Finally, it is essential to remember that content validity as rated by experts is no guarantee of the effectiveness of a set of intervention activities. Researchers and intervention developers should not substitute content validity assessment for rigorous assessment of intervention effectiveness.

### Conclusions

Content validity assessment can be a helpful checkpoint in the process of developing or improving an eHealth intervention. Our team created and implemented a straightforward method and Content Validity Survey Tool that provided useful information regarding the match of intervention activities to underlying program theory. This approach could be appropriately utilized at multiple points during the development process to guide ongoing optimization of eHealth interventions.

## Multimedia Appendix 1

Content validity survey tool.

## Abbreviations

I-CVI: Item-level Content Validity Index
S-CVI/AV: Scale-level Content Validity Index / Average (averaged across I-CVIs)
