# Supplementary material for: A New Method for Assessing Content Validity in Model-Based Creation and Iteration of eHealth Interventions
Source: J Med Internet Res. 2015 Apr 15;17(4):e95. doi: 10.2196/jmir.3811 (PMC4414959; doi:10.2196/jmir.3811)
Supplement: Supplementary file 1 [file jmir_v17i4e95_app1.pdf]

CONTENT VALIDITY SURVEY TOOL

Kassam-Adams et al, Children's Hospital of Philadelphia, 2012

Thank you for agreeing to serve as an expert reviewer for *[NAME OF INTERVENTION]*. We greatly appreciate your contribution to the development of this eHealth intervention. Your input on this survey will help us to evaluate the content validity of the intervention – the extent to which intervention materials and activities match our program theory.

Overview of *[NAME OF INTERVENTION]*

[TEXT OVERVIEW HERE]

See *[DESCRIPTION / FIGURE]* below which depicts our overall program theory / model of change for this eHealth intervention. In this model, INTERVENTION ACTIVITIES are designed to address specific INTERVENTION TARGETS and thereby affect both observable proximal goals (e.g. *[NAME RELEVANT OBSERVABLE BEHAVIORAL GOALS]*), and ultimately the desired health outcomes addressed by this intervention (e.g., *[NAME DESIRED HEALTH OUTCOMES FOR THIS eHEALTH INTERVENTION]*).

Program Theory / Model of Change

[INSERT FIGURE OR OTHER DESCRIPTION OF PROGRAM THEORY / MODEL OF CHANGE UPON WHICH THE INTERVENTION IS BASED]

Expert Survey Directions

RATINGS FOR EACH INTERVENTION ACTIVITY:

For purposes of this survey, we have identified discrete INTERVENTION ACTIVITIES which are paired with the specific INTERVENTION TARGET(S) they address.

We are asking that you make *[NUMBER]* **sets of ratings**.

Please use the 1-4 scales to evaluate each INTERVENTION ACTIVITY on these criteria:

RELEVANCE to the proposed program theory: extent to which each specific intervention activity is pertinent to the intended INTERVENTION TARGET.

EFFECTIVENESS of intervention activities: likelihood that each specific activity will successfully modify the intended INTERVENTION TARGET.

APPROPRIATENESS FOR *[INTENDED USER POPULATION]*: extent to which language, nature of activities, instructions, response choices, etc are clear, easy to understand, and *[CULTURALLY / DEVELOPMENTALLY]* appropriate for *[INTENDED USER POPULATION]*.

Additional Comments:

There is room in each section for any additional comments -- we welcome feedback or suggestions based upon your expertise. Feedback on the intervention as a whole is also welcome.

QUESTIONS?

Please address any questions to *[CONTACT NAME, ROLE, CONTACT DETAILS]*

THANK YOU

[NOTE: FOR EACH ACTIVITY OR SET OF ACTIVITIES, PROVIDE INFORMATION FOR REVIEWERS ABOUT WHERE ACTIVITY CAN BE OBSERVED / REVIEWED, INCLUDING SCREEN SHOT OR OTHER GUIDANCE AS APPROPRIATE]

Activity 1: *[SPECIFIC DESCRIPTION OF ACTIVITY*]

Intended Intervention Target: ***[SPECIFIC DESCRIPTION OF TARGET*]**

Level of **Relevance** to intended Intervention Target	Irrelevant/ Extraneous to this target	1	2	3	4	Central/Key/Essential to this target
Level of likely **Effectiveness** in modifying intended Target	Not likely<br>to be effective	1	2	3	4	Very likely<br>to be effective
**Appropriateness** for *[INTENDED USER POPULATION]*	Content / language / nature of activities inappropriate/ unsuitable for *[INTENDED USER POPULATION]*	1	2	3	4	Content / language / nature of activities appropriate and suitable for *[INTENDED USER POPULATION]*

Comments/Suggestions regarding this intervention activity:

Activity 2, etc.
[REPEAT FOR EACH INTERVENTION ACTIVITY / TARGET PAIRING]

[AT END OF SURVEY]
Please feel free to provide any overall comments / suggestions regarding this intervention:

EXAMPLE ITEM: Adapted from Content Validity Survey Tool for Coping Coach intervention
NOTE: The original Coping Coach Content Validity Survey Tool utilized a 5-point (0 to 4) rating scale for each item.

Section: The Airship

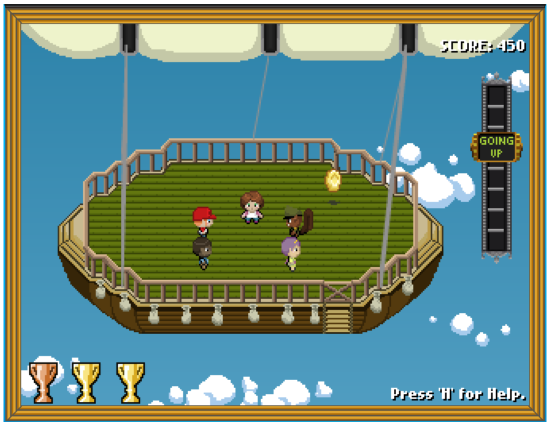

Skill Practice Activity 5:
Player helps Jack and Jayla understand their thoughts and feelings and then helps Jack/Jayla change unhelpful thoughts in order help them to feel better *(See Coping Coach Script- section III, pp. 11-12, lines 34-98)*

Intended Intervention Target (CR3): Child will identify helpful/unhelpful trauma-related appraisals

Level of **Relevance** to intended Intervention Target	Irrelevant/ Extraneous to this target	1	2	3	4	Central/Key/Essential to this target
Level of likely **Effectiveness** in modifying intended Target	Not likely to be effective	1	2	3	4	Very likely to be effective
**Appropriateness** for children ages 8-12 yrs.	Content / language / nature of activities inappropriate/ unsuitable for 8-12	1	2	3	4	Language / nature of activities appropriate and suitable for 8-12

Intended Intervention Target (CR4): Child will utilize cognitive restructuring to modify unhelpful appraisals.

Level of **Relevance** to intended Intervention Target	Irrelevant/ Extraneous to this target	1	2	3	4	Central/Key/Essential to this target
Level of likely **Effectiveness** in modifying intended Target	Not likely to be effective	1	2	3	4	Very likely to be effective
**Appropriateness** for children ages 8-12 yrs.	Content / language / nature of activities inappropriate/ unsuitable for 8-12	1	2	3	4	Language / nature of activities appropriate and suitable for 8-12

Comments/Suggestions regarding this intervention activity:
